# High-Performance NiO/TiO_2_/ZnO Photovoltaic UV Detector

**DOI:** 10.3390/s23052741

**Published:** 2023-03-02

**Authors:** Guoxin Shang, Libin Tang, Gang Wu, Shouzhang Yuan, Menghan Jia, Xiaopeng Guo, Xin Zheng, Wei Wang, Biao Yue, Kar Seng Teng

**Affiliations:** 1 School of Materials and Energy, Yunnan University, Kunming 650500, China; 2 Kunming Institute of Physics, Kunming 650223, China; 3 Yunnan Key Laboratory of Advanced Photoelectric Materials and Devices, Kunming 650223, China; 4 School of Physics and Astronomy, Yunnan University, Kunming 650500, China; 5 Department of Electronic and Electrical Engineering, Swansea University, Bay Campus, Fabian Way, Swansea SA1 8EN, UK

**Keywords:** NiO, TiO_2_, ZnO, heterojunction, UV detector

## Abstract

The ultraviolet (UV) photodetector has found many applications, ranging from optical communication to environmental monitoring. There has been much research interest in the development of metal oxide-based UV photodetectors. In this work, a nano-interlayer was introduced in a metal oxide-based heterojunction UV photodetector to enhance the rectification characteristics and therefore the device performance. The device, which consists of nickel oxide (NiO) and zinc oxide (ZnO) sandwiching an ultrathin dielectric layer of titanium dioxide (TiO_2_), was prepared by radio frequency magnetron sputtering (RFMS). After annealing, the NiO/TiO_2_/ZnO UV photodetector exhibited a rectification ratio of 10^4^ under UV irradiation of 365 nm at zero bias. The device also demonstrated a high responsivity of 291 A/W and a detectivity of 6.9 × 10^11^ Jones at +2 V bias. Such a device structure provides a promising future for metal oxide-based heterojunction UV photodetectors in a wide range of applications.

## 1. Introduction

The ultraviolet (UV) photoelectric detector has attracted much research interest, as it has found many important applications in optical communication, remote control, astronomical imaging, bioaerosol detection, fire detection, and environmental monitoring [1,2,3,4,5,6]. In recent years, there has been much research on the use of wide band gap metal oxide semiconductor materials, such as NiO, ZnO, TiO_2_, Ga_2_O_3_, SnO_2_, etc., to develop UV photodetectors. These materials offer good stability, rapid response, high sensitivity, and low production cost, which are essential criteria for the applications of UV photodetectors [7,8,9,10].

NiO is a p-type semiconductor material. It has a direct band gap of ~3.6–4.3 eV [11], a high melting point of 1960 °C, and a relatively high exciton binding energy of 110 meV [12]. Furthermore, the environmental friendliness and abundance of NiO make it an attractive material in the fabrication of UV photodetectors for widespread applications [13,14,15]. ZnO is an n-type semiconductor material with a band gap energy of ~3.3 eV [16,17]. It is inexpensive, non-toxic, and provides high optical transparency. Moreover, the material can be easily synthesized on an industrial scale [18,19].

There are several reports on the study of UV photodetectors based on NiO/ZnO heterojunction. For example, Rana [20] et al. fabricated an Ag-NWs/NiO film/ZnO film/FTO UV detector, and the responsivity of the device was 0.29 A/W. Zhang [21] and Shen [22] prepared Cr/Au/NiO nanofiber/ZnO nanofiber/Al and Ag/ZnO nanorod/NiO film/FTO UV detectors and the responsivities of these devices were 0.42 and 0.44 mA/W, respectively. However, these UV photodetectors based on NiO/ZnO heterojunction exhibited relatively poor photo-response and device performance. Jia [23] et al. reported a p-NiO/SiO_2_/n-ZnO ultraviolet detector, with the device showing high performance with responsivity and detectivity of 5.77 A/W and 1.51 × 10^11^ Jones, respectively. To obtain a higher performance of the UV photovoltaic detector, TiO_2_ is used instead of SiO_2_. Adding a dielectric layer is equivalent to adding a barrier in the heterojunction of the device, and different barriers can change the efficiency of the carrier transport [24]. The band gap of TiO_2_ is smaller than that of SiO_2_; meanwhile, TiO_2_ has a larger dielectric constant than SiO_2_, which is easier to excite electrons and more conducive to charge storage.

In this work, an ultra-thin dielectric layer of TiO_2_ was introduced between the NiO and ZnO layers to enhance the performance of the metal oxide-based heterojunction UV photodetector. After annealing, the NiO/TiO_2_/ZnO heterojunctions exhibited good rectification characteristics with a high rectification ratio of 10^4^. The use of the TiO_2_ interlayer and its role was investigated. The photodetector demonstrated low dark current and excellent optical response. Under 365 nm UV illumination, the maximum responsivity and detectivity of the device was 291 A/W and 6.9 × 10^11^ Jones, respectively.

## 2. Materials and Methods

### 2.1. Materials

Quartz substrate, sputtering targets (such as nickel oxide, zinc oxide target, and titanium dioxide), and ITO were purchased from ZhongNuo Advanced Material (Beijing) Technology Co., Ltd. (Beijing, China). The purity of all the target materials was 99.99%.

### 2.2. Device Fabrication

The quartz substrate was cleaned in a mixture of ammonia, hydrogen peroxide, and deionized water (1:1:2) at 80 °C for 1 h, followed by dipping in deionized water for 30 s and then blow-dried with nitrogen. The sputtering power used to deposit NiO, TiO_2_, and ZnO films was 200, 100, and 200 W, for a duration of 3000, 50, and 3000 s, respectively. The sample was annealed at 300 °C for 2 h after deposition of the films. This was then followed by lithography and the deposition of electrical contacts to the device.

### 2.3. Characterization Techniques

X-ray diffraction (XRD, Rigaku D/Max-23) and transmission electron microscopy (TEM, Tecnai G2 F30 Twin) were used to analyze the crystal structure of the deposited films. The thickness of the film was measured by atomic force microscopy (AFM) using Bruker Dimension ICON, and the thickness of the dielectric film in the device was optimized based on the device performance. A scanning electron microscope (SEM, Nova NanoSEM 450) was used to provide a cross-sectional analysis of the deposited films and thickness measurements of different layers in the device. Keysight B1500A was used for electrical characterization of the device. All measurements were conducted at room temperature.

## 3. Results and Discussion

### 3.1. Morphology and Structure of Thin Films

**Figure 1:**
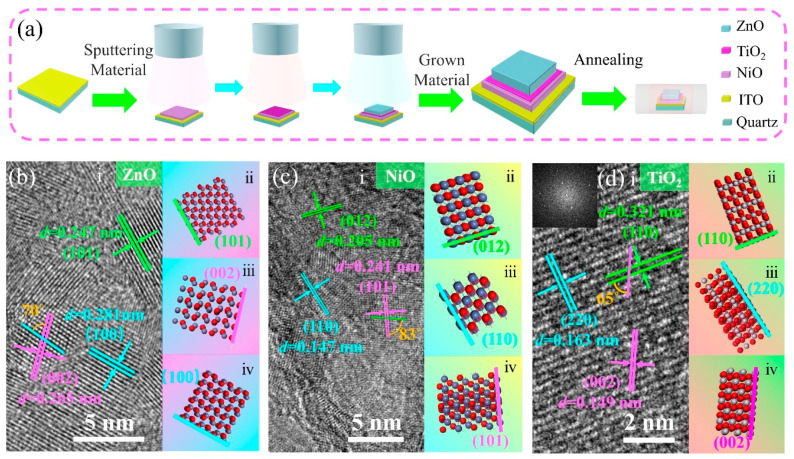
(**a**) Schematic diagram illustrating the preparation process of the films. (**b**–**d**) TEM images of ZnO, NiO, and TiO_2_ (insets show the atomic spatial arrangements of the crystal planes), respectively.

Figure 1a illustrates the preparation process of the films by RFMS. NiO, TiO_2_, and ZnO were deposited successively onto ITO-coated quartz substrate. Figure 1b–d show TEM images of the interplanar spacings of the deposited materials. The interplanar spacing was an average value from 10 lattice fringes measured by Fourier transform. The angle between different crystal faces was obtained directly from the TEM image. Materials Studio software was used to model the crystal structures. ZnO exhibited lattice spacings of 0.247, 0.281, and 0.265 nm that corresponded to its (101), (100), and (002) planes, respectively, as shown in Figure 1b. Furthermore, the angle between the (100) and (002) planes was 70°. Figure 1c shows the lattice spacings of NiO, with 0.205, 0.241, and 0.147 nm corresponding to its (012), (101), and (110) planes, respectively. The angle between the (101) and (110) planes of NiO was 83°. The lattice spacings of TiO_2_ were 0.321, 0.163, and 0.149 nm corresponding to the (110), (220), and (002) planes, respectively, as shown in Figure 1d. It belonged to the rutile-type indices of crystal face, indicating that the prepared TiO_2_ exhibited good crystal quality in the rutile-type phase. The angle between the (110) and (002) planes of TiO_2_ was 65°. All interplanar angles are consistent with those reported in the literature. The atomic spatial arrangements of the crystal planes for the different materials are shown as insets ⅱ, ⅲ, and ⅳ in Figure 1b–d.

**Figure 2:**
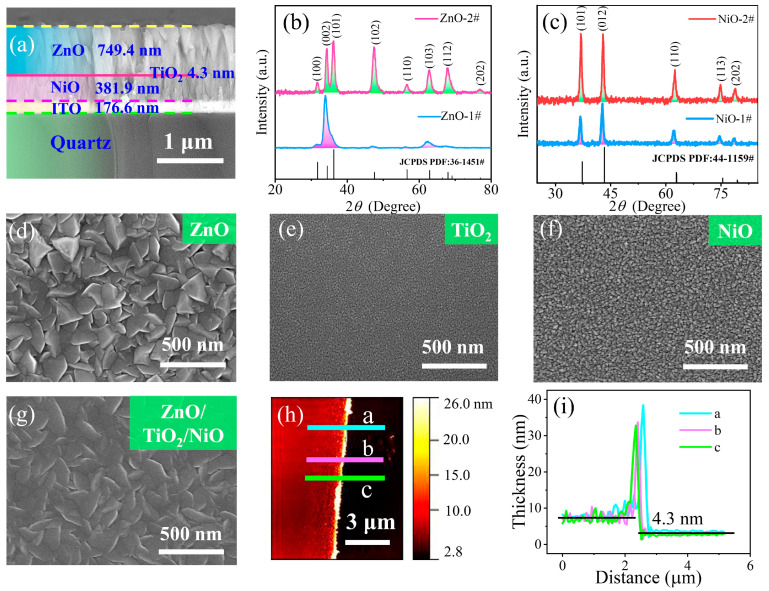
(**a**) Cross-sectional SEM image of ZnO/TiO_2_/NiO films. (**b**,**c**) XRD patterns of ZnO and NiO films, respectively. (**d**–**f**) SEM images of ZnO, TiO_2_, and NiO films, respectively. (**g**) Top-view SEM image of ZnO/TiO_2_/NiO films. (**h**,**i**) AFM image and line profiles for thickness measurement of TiO_2_ film, respectively.

Figure 2a shows the cross-sectional SEM image of the deposited films. The film thickness, which has an important influence on the device performance, can be deduced from the image. From top to bottom, the image reveals the deposited films of ZnO, TiO_2_, NiO, and ITO having a thickness of ~749.4, 4.3, 381.9, and 176.6 nm, respectively, on the quartz substrate. The cross-sectional image also shows that the different metal-oxide films exhibited longitudinally dense growth mode. Figure 2b shows the XRD patterns of the ZnO film before (ZnO-1#) and after (ZnO-2#) annealing at 300 °C for 2 h. Compared with the standard PDF cards, peaks related to the (002) and (103) crystal planes were observed before annealing of the ZnO film. After annealing, an increase in the number of crystal planes was evident. The full width at half maximum (FWHM) of the (002) crystal plane was significantly narrowed after annealing. This indicates that the annealing of the ZnO film has led to a significant reduction in the defect density and improvement in the crystal quality, which is favorable to the transport of charge carriers in the material. Figure 2c shows the XRD patterns of the NiO film before (NiO-#1) and after (NiO-#2) annealing at 300 °C for 2 h. It was found that the number of NiO diffraction patterns remained the same before and after annealing. There were five crystal planes, namely, (101), (012), (110), (113) and (202), related to the NiO film (similar to the standard PDF cards). However, the intensity of all diffraction patterns became stronger after annealing, and the FWHM of these patterns was significantly narrowed. The diffraction patterns in Figure 2b,c were consistent with the observed crystal planes in the TEM images in Figure 1b,c. Figure 2d–f show the SEM images on the surface morphology of the three metal oxide films. Large particles with obvious boundaries were observed in the ZnO film. NiO particles were relatively smaller than those of ZnO but exhibited tapered features. The TiO_2_ film has much finer and denser particles with good size distribution. An SEM image of the top view of the ZnO/TiO_2_/NiO films is shown in Figure 2g. The grain boundary became less obvious after annealing. Figure 2h shows an AFM image of the TiO_2_ film with a step edge. The line profiles taken across the step edge are shown in Figure 2i. From the line profiles, the thickness of the TiO_2_ film was measured to be ~4.3 nm, which is consistent with the thickness measured in Figure 2a.

### 3.2. Device Performance and Analysis

**Figure 3:**
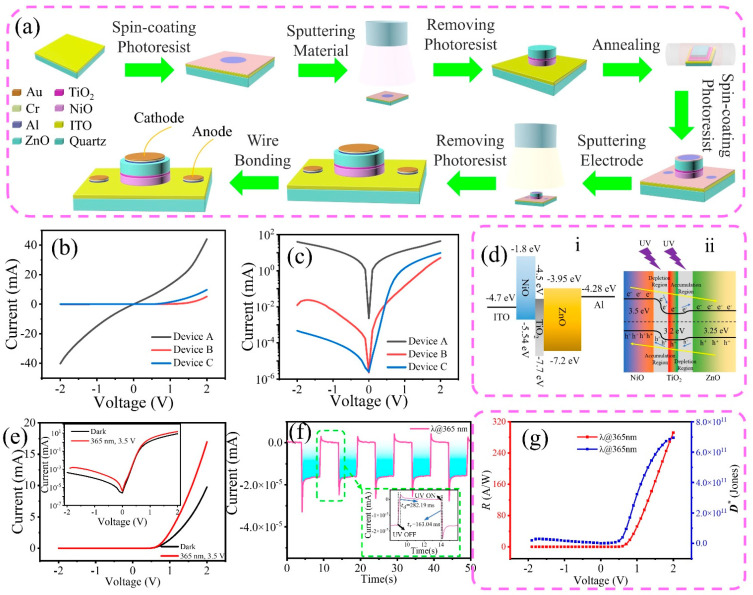
(**a**) Schematic diagram depicting the fabrication process of the UV photodetector. (**b**,**c**) *I*−*V* characteristics and logarithmic plots of Device ‘A’, ‘B’, and ‘C’, respectively. (**d**) Energy band diagram of the metal oxide-based heterojunction UV photodetector. (**e**) *I*−*V* plots of Device ‘C’ in the dark and under 365 nm illumination, represented by black and red curves, respectively (inset shows the logarithmic *I*−*V* plot). (**f**) Switching behavior of Device ‘C’ under 365 nm illumination (inset shows a magnified period). (**g**) Responsivity (red) and detectivity (blue) plots of Device ‘C’.

Figure 3a depicts the fabrication process of the UV photodetector. Figure 3b,c show the *I*−*V* characteristics of three different devices, namely, Device ‘A’, ‘B’ and ‘C’. Device ‘A’ (represented by black curves) consisted of the NiO/ZnO heterojunction and did not exhibit rectification behavior. Device ‘B’ (represented by red curves), which consisted of an ultra-thin TiO_2_ dielectric layer between the NiO and ZnO films, exhibited slight rectification behavior. Device ‘C’ (represented by blue curves) has the same device structure as Device ‘B’ but has undergone annealing treatment. It demonstrated very good rectification characteristics with an excellent rectification ratio of 10^4^. The results showed that the rectification characteristic of the device was significantly improved by introducing a dielectric inter-layer and annealing treatment. Figure 3d shows the energy band structure of the metal oxide-based UV photodetector. Photogenerated charge carriers are generated under UV light irradiation and driven by an external electric field, causing electrons to flow from NiO to ZnO while holes flow in the opposite direction. The role of the TiO_2_ is to block the migration of charge carriers, hence improving the rectification characteristic of the device. Figure 3e shows the *I*−*V* characteristics of Device ‘C’ under dark condition and 365 nm light with a power density of 1.479 mW/cm^2^. When the UV light was switched on or off, the device exhibited good stability and repeatability. When the UV light was turned on (off), the current of the device changed rapidly, indicating that the heterojunction device has a fast UV response. The device has a rise time and a fall time (defined as the time required for the photocurrent to increase from 10% to 90% and drop from 90% to 10%) of 163.04 and 282.19 ms, respectively, at 0 V bias, as shown in Figure 3f. Responsivity (*R*) and detectivity (D*) are important performance parameters for a photodetector. They are calculated using the followings formulae [25]:

(1) $$R=\frac{I_{ph}-I_{d}}{PA}$$

(2) $$D^{*}=\frac{R}{\sqrt{2qI_{d}/A}}$$

where *I_ph_* and *I_d_* are photocurrent and dark current, respectively, *P* is optical power density, *A* is effective irradiation area, and q is unit charge. The plots of responsivity and detectivity of the photodetector in this study are shown in Figure 3g. The maximum value of the responsivity and detectivity of the device was 291 A/W and 6.9 × 10^11^ Jones, respectively, comparable with the NiO/ZnO-based heterojunction UV detector, as shown in Table 1.

## 4. Conclusions

In this work, a metal oxide-based heterojunction UV photodetector consisting of NiO/TiO_2_/ZnO was prepared via RFMS. The implementation of a TiO_2_ dielectric layer between the NiO and ZnO films and annealing treatment significantly enhanced the rectification characteristic of the device. This led to a reduction in the dark current and an improvement in the optical response of the device. The device demonstrated a rectification ratio of 10^4^, and a rapid response under 365 nm UV illumination, with a maximum responsivity and detectivity of 291 A/W and 6.9 × 10^11^ Jones, respectively.

## Figures and Tables

**Table 1:** Performance parameters of UV detector based on NiO/ZnO.

Detector	Wavelength (nm)	*R*	D* (Jones)	Ref.
NiO/ZnO	365	0.29 A/W	2.75 × 10^11^	[20]
NiO/ZnO	350	0.415 mA/W	-	[21]
NiO/ZnO	355	0.44 mA/W	-	[22]
NiO/ZnO	370	0.14 A/W	2.9 × 10^12^	[26]
NiO/ZnO	365	13.01 mA/W	5.66 × 10^11^	[27]
NiO/ZnO	365	2.23 mA/W	-	[28]
NiO/TiO_2_/ZnO	365	291 A/W	6.9 × 10^11^	This work

## Data Availability

Not applicable.

## References

[1] Zhao B., Wang F., Chen H., Zheng L., Su L., Zhao D., Fang X. (2017). An Ultrahigh Responsivity (9.7 mA W^−1^) Self-Powered Solar-Blind Photodetector Based on Individual ZnO-Ga_2_O_3_ Heterostructures. Adv. Funct. Mater..

[2] Chen H., Liu H., Zhang Z., Hu K., Fang X. (2016). Nanostructured Photodetectors: From Ultraviolet to Terahertz. Adv. Mater..

[3] Li L., Lee P.S., Yan C., Zhai T., Fang X., Liao M., Koide Y., Bando Y., Golberg D. (2010). Ultrahigh-Performance Solar-Blind Photodetectors Based on Individual Single-crystalline In_2_Ge_2_O_7_ Nanobelts. Adv. Mater..

[4] Lyu C.G., Tian J., Wen-bo Y., Tian Q.J., Ying-hao L., Liu Z.M., Han-mo Z. (2016). Solar-Blind Ultraviolet Upwelling Radiance Diurnal Variation Led by Observation Geometry Factors on Geostationary Attitude Sensor Limb Viewing. Terr. Atmos. Ocean. Sci..

[5] Wang T.J., Xu W.Z., Lu H., Ren F.F., Chen D.J., Zhang R., Zheng Y.D. (2014). Solar-blind ultraviolet band-pass filter based on metal-dielectric multilayer structures. Chin. Phys. B.

[6] Qian L.X., Wang Y., Wu Z.H., Sheng T., Liu X.Z. (2017). Beta-Ga_2_O_3_ solar-blind deep-ultraviolet photodetector based on annealed sapphire substrate. Vacuum.

[7] Wei C., Xu J., Shi S., Bu Y., Cao R., Chen J., Xiang J., Zhang X., Li L. (2020). The improved photoresponse properties of self-powered NiO/ZnO heterojunction arrays UV photodetectors with designed tunable Fermi level of ZnO. J. Colloid Interface Sci..

[8] Zu X., Wang H., Yi G., Zhang Z., Jiang X., Gong J., Luo H. (2015). Self-powered UV photodetector based on heterostructured TiO_2_ nanowire arrays and polyaniline nanoflower arrays. Synth. Met..

[9] Lin Z.H., Cheng G., Yang Y., Zhou Y.S., Lee S., Wang Z.L. (2014). Triboelectric Nanogenerator as an Active UV Photodetector. Adv. Funct. Mater..

[10] He Y., Zhang W., Zhang S., Kang X., Peng W., Xu Y. (2012). Study of the photoconductive ZnO UV detector based on the electrically floated nanowire array. Sens. Actuators A Phys..

[11] Jiang F., Choy W.C., Li X., Zhang D., Cheng J. (2015). Post-treatment-Free Solution-Processed Non-stoichiometric NiOx Nanoparticles for Efficient Hole-Transport Layers of Organic Optoelectronic Devices. Adv. Mater..

[12] Mohanty P., Rath C., Mallick P., Biswal R., Mishra N.C. (2010). UV-visible studies of nickel oxide thin film grown by thermal oxidation of nickel. Phys. B Condens. Matter.

[13] Patel M., Kim H.S., Kim J. (2015). All Transparent Metal Oxide Ultraviolet Photodetector. Adv. Electron. Mater..

[14] Manders J.R., Lai T.H., An Y., Xu W., Lee J., Kim D.Y., Bosman G., So F. (2014). Low-Noise Multispectral Photodetectors Made from All Solution-Processed Inorganic Semiconductors. Adv. Funct. Mater..

[15] Kim D.Y., Ryu J., Manders J., Lee J., So F. (2014). Air-Stable, Solution-Processed Oxide p-n Heterojunction Ultraviolet Photodetector. ACS Appl. Mater. Interfaces.

[16] Lee K.M., Lai C.W., Ngai K.S., Juan J.C. (2016). Recent developments of zinc oxide based photocatalyst in water treatment technology: A review. Water Res..

[17] Son D.Y., Im J.H., Kim H.S., Park N.G. (2014). 11% Efficient Perovskite Solar Cell Based on ZnO Nanorods: An Effective Charge Collection System. J. Phys. Chem. C.

[18] Wang J., Chen R., Xiang L., Komarneni S. (2018). Synthesis, properties and applications of ZnO nanomaterials with oxygen vacancies: A review. Ceram. Int..

[19] Abbasi M.A., Ibupoto Z.H., Khan A., Nur O., Willander M. (2013). Fabrication of UV photo-detector based on coral reef like p-NiO/n-ZnO nanocomposite structures. Mater. Lett..

[20] Rana A.K., Kumar M., Ban D.K., Wong C.P., Yi J., Kim J. (2019). Enhancement in Performance of Transparent p-NiO/n-ZnO Heterojunction Ultrafast Self-Powered Photodetector via Pyro-Phototronic Effect. Adv. Electron. Mater..

[21] Zhang Z., Ning Y., Fang X. (2019). From nanofibers to ordered ZnO/NiO heterojunction arrays for self-powered and transparent UV photodetectors. J. Mater. Chem. C.

[22] Shen Y., Yan X., Bai Z., Zheng X., Sun Y., Liu Y., Lin P., Chen X., Zhang Y. (2015). A self-powered ultraviolet photodetector based on solution-processed p-NiO/n-ZnO nanorod array heterojunction. RSC Adv..

[23] Jia M., Wang F., Tang L., Xiang J., Teng K.S., Lau S.P., Lu Y. (2023). Low-power-consumption ultraviolet photodetector based on p-NiO/SiO_2_/ n-ZnO. Opt. Laser Technol..

[24] Kim C., Yoo T.J., Chang K.E., Kwon M.G., Hwang H.J., Lee B.H. (2021). Highly responsive near-infrared photodetector with low dark current using graphene/germanium Schottky junction with Al_2_O_3_ interfacial layer. Nanophotonics.

[25] Cao R., Xu J., Shi S., Chen J., Liu D., Bu Y., Zhang X., Yin S., Li L. (2020). High-performance self-powered ultraviolet photodetectors based on mixed-dimensional heterostructure arrays formed from NiO nanosheets and TiO_2_ nanorods. J. Mater. Chem. C.

[26] Hasan M.R., Xie T., Barron S.C., Liu G., Nguyen N.V., Motayed A., Rao M.V., Debnath R. (2015). Self-powered p-NiO/n-ZnO heterojunction ultraviolet photodetectors fabricated on plastic substrates. APL Mater..

[27] Salunkhe P., Bhat P., Kekuda D. (2022). Performance evaluation of transparent self-powered n-ZnO/p-NiO heterojunction ultraviolet photosensors. Sens. Actuators A Phys..

[28] Long H., Ai L., Li S., Huang H., Mo X., Wang H., Chen Z., Liu Y., Fang G. (2014). Photosensitive and temperature-dependent I-V characteristics of p-NiO film/n-ZnO nanorod array heterojunction diode. Mater. Sci. Eng. B.

